# Carriage of multidrug-resistant bacteria and encoding genes among Vietnamese children with acute diarrhea

**DOI:** 10.1016/j.ijregi.2026.100844

**Published:** 2026-01-12

**Authors:** Xuan Duong Tran, Thi Loi Dao, Ndiaw Goumballa, Trong Kiem Tran, Thanh Binh Nguyen, Duy Cuong Nguyen, Pierre Marty, Philippe Gautret

**Affiliations:** 1 Aix Marseille Univ, AP-HM, SSA, RITMES, Marseille, France; 2 Institut Hospitalo-Universitaire Méditerranée Infection, Marseille, France; 3 Thai Binh University of Medicine and Pharmacy, Hung Yen, Vietnam; 4 Thai Binh Pediatric Hospital, Hung Yen, Vietnam; 5 Université Côte D'Azur, Inserm, C3M, Nice, France; 6 Parasitologie-Mycologie, Centre Hospitalier Universitaire L'Archet, Nice, France

**Keywords:** Acute diarrhea, Children, Antibiotic resistant, Multidrug-resistant, MRSA, ESBL

## Abstract

**Objectives:**

This study aimed to assess the prevalence of multidrug-resistant (MDR) bacterial carriage and resistance-encoding genes in children with acute diarrhea.

**Methods:**

A prospective study was conducted from July 2020 to July 2021 in hospitalized children aged under 5 years. Stool samples were collected at hospital admission. MDR bacteria were identified by culture, antimicrobial susceptibility patterns were assessed using the disk diffusion method, and resistance genes were investigated in isolates, using real-time polymerase chain reaction. Colistin resistance genes were also tested from stool samples.

**Results:**

A total of 451 children were included; 33.2% harbored at least one MDR bacteria and/or one colistin resistance genes. A total of 79 (17.6%) children were positive for at least one MDR pathogen by culture. A total of 100 isolates were identified, including 17 methicillin-resistant *Staphylococcus aureus* and 83 Enterobacteriaceae. *Escherichia coli* was the most common (11.8%), followed by *S. aureus* (3.8%) and *Klebsiella pneumoniae* (2.7%). High resistance levels were observed in tested isolates: 94.8% to ceftriaxone, 77.1% to cefepime, 42.2% to piperacillin–tazobactam, and 19.3% to ertapenem. Among methicillin-resistant *S. aureus* isolates, 15 of 17 (88.2%) carried the *mecA* gene. Among Enterobacteriaceae, 73/83 (88.0%) carried *bla_CTX-M-A_*, 49 (59.0%) had *bla_TEM_*, 19 (22.9%) had *bla_SHV_*, and six (7.2%) were positive for *bla_NDM_*. In addition, 87 (19.3%) of 451 children carried at least one colistin resistance gene, with 10.9% of *mcr-1*.

**Conclusions:**

MDR bacterial carriage and resistance genes were frequently detected at hospital admission, reflecting community or previous health care–associated exposure rather than in-hospital antibiotic selection. These findings underscore the urgent need for strengthened antimicrobial stewardship, rational antibiotic use, improved infection control, and enhanced diagnostic capacity.

## Highlights

• This work investigated the phenotypic and genetic features of multidrug-resistant (MDR) bacteria.
• A total of 33.2% children with acute diarrhea harbored at least one MDR bacteria and/or one colistin-resistance genes.
• Extended-spectrum β-lactamase–producing *Escherichia coli* was the predominant MDR pathogen.
• *bla_CTX-M-A_* was the most frequent resistance gene among Enterobacteriaceae.
• Colistin resistance genes, especially *mcr-1* and *mcr-3*, were commonly detected.

## Introduction

The emergence and spread of multidrug-resistant bacteria (MDR) present a significant threat to public health worldwide, constituting a critical challenge in the management of infectious diseases [1]. MDR bacteria, defined as pathogens resistant to multiple classes of antimicrobial agents, significantly compromise the effectiveness of antibiotic therapy, leading to prolonged illnesses, treatment failures, increased health care costs, and elevated mortality rates. The most vulnerable demographic groups susceptible to the deleterious effects of MDR infections are children [2].

Indeed, children represent a particularly vulnerable population due to the unique characteristics of their physiology and behavior. Their developing immune systems, although resilient, are not fully matured, rendering them more susceptible to infections and less able to combat antibiotic-resistant pathogens effectively [3]. Moreover, children often engage in behaviors and activities that increase their exposure to infectious agents, such as attending daycare centers, schools, and recreational facilities, where close contact with other children facilitates the transmission of bacteria and resistance genes [4,5]. In addition, frequent visits to health care settings for routine checkups, vaccinations, and treatment of illnesses expose children to environments where MDR bacteria are prevalent, further amplifying their risk of acquiring resistant infections [2,6,7].

Antibiotic resistance is a complex phenomenon driven by various factors, including antibiotic overuse, inadequate infection control practices, and the horizontal transfer of resistance genes among bacterial populations [1,8]. Understanding the prevalence and distribution of MDR bacteria and the genetic determinants conferring resistance is crucial for devising effective strategies to mitigate the spread of antibiotic resistance and optimize treatment outcomes [[8], [9], [10]]. Moreover, elucidating the dynamics of MDR acquisition among children can inform targeted interventions to reduce transmission and preserve the efficacy of antibiotics, particularly, in resource-limited settings such as Vietnam [11,12].

In the context of Vietnam, a rapidly developing country characterized by urbanization and evolving health care infrastructure, the burden of MDR infections among children is a pressing concern [13,14]. The dynamic socio-economic landscape, coupled with the increasing accessibility and use of antibiotics, has fueled the emergence and dissemination of MDR bacteria in community and health care settings [6,7,15,16]. Furthermore, challenges in infection control practices, limited access to diagnostic tools, and suboptimal antibiotic prescribing practices contribute to the amplification of antibiotic resistance in community and health care settings [[8], [9], [10]]. Therefore, understanding the epidemiology, transmission dynamics, and genetic determinants of MDR bacteria among Vietnamese children is paramount for devising targeted interventions to mitigate the spread of antibiotic resistance and improve treatment outcomes.

In this study, we conducted an investigation of MDR enteric bacteria and resistance-encoding genes among Vietnamese children suffering diarrhea, leveraging phenotypic and genotypic approaches. We aim to assess the prevalence of MDR infections and characterized the antimicrobial susceptibility profiles of the isolated pathogens. Furthermore, we used molecular techniques to elucidate the genetic mechanisms underlying antibiotic resistance and track the dissemination of resistance genes within bacterial populations. The findings generated from this research have the potential to inform evidence-based strategies for antimicrobial stewardship, infection prevention, and therapeutic interventions tailored to the unique challenges posed by antibiotic resistance in pediatric health care settings.

## Methods

### Study design

This prospective study was carried out at Thai Binh Pediatric Hospital (TBPH).

Participants were eligible if they were younger than 5 years of age and were admitted to the gastroenterology department with a diagnosis of acute diarrhea, defined as the occurrence of loose, watery, or bloody stools three or more times within 24 hours. Stool specimens were collected at hospital admission, before initiation of in-hospital antibiotic treatment. One stool sample was collected per participant. The target organisms were multidrug-resistant bacteria, including methicillin-resistant *Staphylococcus aureus* (MRSA), extended-spectrum β-lactamase (ESBL)–producing Enterobacterales (ESBL-E)–producing Enterobacterales, carbapenem-resistant Gram-negative bacteria, and colistin resistance genes.

### Setting

TBPH is the principal pediatric referral center in Thai Binh Province, admitting around 2000 children each month. This is a main provincial pediatric referral hospital in northern Vietnam, providing care to urban and rural populations. However, its capacity for microbiological testing remains limited [17,18].

The clinical setting included children admitted to the gastroenterology department with acute diarrhea.

### Specimen types and sampling period

Stool specimens were collected after each child passed a bowel movement at admission (day 0), after written informed consent was obtained from parents or legal guardians, then placed into Sigma-Transwab transport medium. The samples were collected once per participant over a 12-month period of study, from July 2020 to July 2021. Within 48 hours of collection, the samples were delivered to the laboratory at TBPH and preserved at −80°C. Subsequently, all specimens were transferred on dry ice to the Institut Hospitalo-Universitaire Méditerranée Infection in Marseille, France for further analysis.

### Laboratory work

#### Identification of multidrug-resistant bacteria

The etiology of diarrhea among studied population was investigated and published elsewhere [17]. In this study, we only focus on multidrug-resistant bacteria identification.

MRSA was detected using MRSA agar (bioMérieux, Marcy l’Étoile, France). MacConkey agar (bioMérieux) was used to recover ESBL-E, carbapenem-resistant *Acinetobacter baumannii*, and *Pseudomonas aeruginosa* isolates resistant to ceftazidime or cefepime. Screening for carbapenemase-producing Enterobacterales and glycopeptide-resistant *Enterococcus spp.* was performed using SMART agar and vancomycin-resistant Enterococcus agar (bioMérieux), respectively.

All MDR organisms grown on culture were subjected to identification and antimicrobial susceptibility testing. Bacterial species were confirmed by matrix-assisted laser desorption/ionization–time-of-flight mass spectrometry (Microflex, Bruker Daltonics, Bremen, Germany). Antimicrobial susceptibility patterns were assessed using the disk diffusion method, and results were interpreted according to the European Committee on Antimicrobial Susceptibility Testing version 6.0 guidelines (http://www.eucast.org). When European Committee on Antimicrobial Susceptibility Testing break points were unavailable, French national recommendations were applied (https://www.sfm-microbiologie.org/wp-content/uploads/2019/02/CASFM-V2.0.Mai2017.pdf).

ESBL-producing Enterobacterales were evaluated against a panel of 13 antibiotics: amoxicillin, amoxicillin–clavulanic acid, piperacillin–tazobactam, cefepime, ceftriaxone, ertapenem, imipenem, fosfomycin, sulfamethoxazole–trimethoprim, gentamicin, ciprofloxacin, doxycycline, and amikacin.

Carbapenem-resistant *A. baumannii* isolates were tested for susceptibility to 14 agents, including ticarcillin, ticarcillin–clavulanic acid, piperacillin–tazobactam, ceftazidime, cefepime, imipenem, doripenem, fosfomycin, fusidic acid, sulfamethoxazole–trimethoprim, tobramycin, ciprofloxacin, colistin, and amikacin.

MRSA isolates underwent susceptibility testing to 13 antibiotics: oxacillin, rifampicin, clindamycin, erythromycin, pristinamycin, gentamicin, vancomycin, teicoplanin, doxycycline, fosfomycin, ciprofloxacin, fusidic acid, and sulfamethoxazole–trimethoprim.

Enterococcus strains were examined against 11 antibiotics, including penicillin G, rifampicin, clindamycin, erythromycin, pristinamycin, gentamicin, vancomycin, teicoplanin, doxycycline, fosfomycin, and fusidic acid.

#### Detection of antibiotic resistance-encoding genes

Antibiotic resistance genes were investigated in all isolates obtained from culture. Genomic DNA was extracted using the QIAamp DNA Mini Kit (Qiagen) on the EZ1 Advanced XL platform (Qiagen), following the manufacturer’s protocol.

For MRSA isolates, the presence of *mecA* and *mecC* genes was assessed to confirm methicillin resistance. In ESBL-producing Enterobacterales and *Pseudomonas aeruginosa*, resistance determinants were identified by targeting bla_CTX-M-A_ (groups CTX-M-1 and CTX-M-9), *bla_CTX-M-B_* (groups CTX-M-2, -M-8, and -M-25), *bla_SHV_, and bla_TEM_*. Carbapenem-resistant *A. baumannii* isolates were screened for *bla_OXA-23_, bla_OXA-24_, bla_OXA-58_*, and *bla_NDM_*. Screening for carbapenemase-producing Enterobacterales included amplification of *bla_OXA-48_, bla_NDM_, bla_VIM_, bla_IMP_*, and *bla_KPC_*.

Each polymerase chain reaction (PCR) run incorporated negative controls (PCR master mix combined with sterile distilled water) and positive controls. A gene target was considered present when the amplification cycle threshold value was ≤26.

Also, we screened for colistin resistance genes by real-time PCR, performed directly from stool samples. Results were considered positive when the cycle threshold value of real-time PCR was <35. The qPCR amplification was used to confirm the presence of *mcr-1, mcr-2* (including the detection of *mcr-6*), *mcr-3, mcr-4, mcr-5, mcr-8, mcr-9*, and *mcr-10* genes using primers previously described.

The identification of MDR bacteria and antibiotic-resistant encoding genes were performed followed the method described in a previous work [19].

#### Quality assurance

All microbiological analyses were performed at the Institut Hospitalo-Universitaire Méditerranée Infection laboratory, which follows standardized operating procedures and participates in internal and external quality control programs for bacterial identification and antimicrobial susceptibility testing.

#### Bias

To minimize selection and duplication bias, only one stool sample was collected per participant at admission. When more than one MDR organism was isolated from the same sample, each isolate was reported separately. However, patients were counted only once for MDR carriage. No sequential samples were collected; therefore, temporal changes in carriage could not be assessed.

### Statistical analysis

STATA software version 15.1 (Copyright 1985-2017 StataCorp LLC, http://www.stata.com, College Station, TX, USA) was used for statistical analysis. No comparative analysis was conducted. The qualitative variables were presented as absolute numbers and percentages.

## Results

### Characteristics of studied population

During the study period, 451 children with acute diarrhea were included. The characteristics of the study population have been described in detail elsewhere [17]. In brief, 252 (55.9%) children were under 12 months of age, 183 (40.6%) were between 12 and 36 months, and 16 (3.6%) were between 36 and 59 months. A total of 294 (65.2%) participants were male. Most (408, 90.5%) children had a normal nutritional status, whereas 23 (5.1%) had moderate malnutrition.

Regarding treatment, 437 (96.9%) children received antibiotics; cephalosporins (366, 81.2%) was the most frequently prescribed. A total of 89 (19.7%) children were treated with a combination of antibiotics. Overall, 415 (92.0%) children recovered and were discharged home, whereas 36 (8.0%) were discharged against medical advice. No deaths were reported during the study period.

A proportion of 65.2% (294 of 451) children were positive with at least one virus and 29.3% (132 of 451) were positive with at least one bacterium, identified by real-time PCR. Adenovirus, norovirus, enterovirus, rotavirus, *Campylobacter jejuni*, enteroaggregative *E. coli*, and enteropathogenic *E. coli* were the most frequent, detected in 35.7% (161 of 451), 25.7% (116 of 451), 20.6% (93 of 451), 14.9% (67 of 451), 14.2% (64 of 451), 8.4% (38 of 451), and 2.9% (13 of 451), respectively [17].

### Detection of MDR bacteria by culture

A total of 79 (17.6%) of 451 patients were positive for at least one MDR bacterial pathogen by culture, and 100 MDR isolates were identified by culture. These included 17 MRSA isolates and 83 Enterobacteriaceae isolates. The most frequently isolated MDR organism was *E. coli*, detected in 53 (11.8%) of 451 patients, with 63 corresponding isolates. This was followed by *S. aureus* (17 of 451 patients, 3.8%; 17 isolates), *K. pneumoniae* (12 of 451 patients, 2.7%; 13 isolates), and *Enterobacter cloacae* (two of 451 patients, 0.4%; 3 isolates) (Table 1).

**Table 1:** Multidrug-resistant bacteria isolated using selective culture medium.

Bacteria	Patient		Isolate	
	n	%	n	%
**At least one multidrug-resistant bacteria**	79	17.5	100	100
**Per medium**				
Methicillin-resistant *Staphylococcus aureus*	17	3.8	17	17.0
MacConkey	64	14.2	77	77.0
SMART	5	1.1	6	6.0
Vancomycin-resistant Enterococcus	0	0.0	0	0.0
**Per pathogen**				
*Staphylococcus aureus*	17	3.8	17	17.0
*Escherichia coli*	53	11.8	63	63.0
*Klebsiella pneumoniae*	12	2.7	13	13.0
*Klebsiella variicola*	1	0.2	1	1.0
*Salmonella paratyphi*	1	0.2	1	1.0
*Salmonella typhi*	1	0.2	1	1.0
*Enterobacter cloacae*	2	0.4	3	3.0
*Enterobacter kobei*	1	0.2	1	1.0

The antibiogram of isolated bacteria is presented in Supplementary Tables 1, 2, and 3. Ceftriaxone was resisted in 94.8% (73 of 77) of the tested isolates. The resistance proportion of cefepime, piperacillin–tazobactam, ertapenem, amikacin were 77.1% (64 of 83), 42.2% (35 of 83), 19.3% (16/83), and 8.4% (seven of 83) tested isolates, respectively.

### Antibiotic resistance encoding genes by qPCR

Among the 17 MRSA isolates, 15 (88.2%) carried the *mecA* gene, whereas none were positive for the *mecC* gene (Table 2).

**Table 2:** Antibiotic resistance encoding genes by quantitative polymerase chain reaction in cultivated isolates.

Encoding genes	N	%	Pathogens
**In 17 methicillin-resistant *Staphylococcus aureus* isolates**			
*mecA*	15	88.2	*S. aureus* (15)
*mecC*	0	0	
**In 83 Enterobacteriaceae isolates**			
*bla_CTX-M-A_*	73	88.0	*Enterobacter cloacae* (3) *Enterobacter kobei* (1) *Escherichia coli* (55) *Klebsiella pneumoniae* (11) *Klebsiella veriicola* (1) *Salmonella paratyphi* (1) *Salmonella typhi* (1)
*bla_CTX-M-B_*	0	0	
*bla_SHV_*	19	22.9	*E. cloacae* (1) *E. kobei* (1) *E. coli* (5) *K. pneumoniae* (12)
*bla_TEM_*	49	59.0	*E. cloacae* (2) *E. coli* (39) *K. pneumoniae* (7) *K. veriicola* (1)
*bla_NDM_*	6	7.2	*E. cloacae* (1) *E. coli* (3) *K. pneumoniae* (1) *K. veriicola* (1)
*bla_VIM_*	0	0	
*bla_KPC_*	0	0	
*bla_OXA48_*	0	0	
*bla_OXA24_*	0	0	
*bla_OXA23_*	0	0	

Of the 83 Enterobacteriaceae isolates, 73 (88.0%) harbored the *bla_CTX-M-A_* gene, 49 (59.0%) were positive for *bla_TEM_*, and 19 (22.9%) carried *bla_SHV_*. Only six (7.2%) isolates were positive for the *bla_NDM_* gene (Table 2).

### Screening for colistin-resistance genes

Screening for colistin resistance genes among all 451 patients showed that *mcr-1* was the most frequently detected gene, present in 49 (10.9%) patients. This was followed by *mcr-3* (44 patients, 9.8%), *mcr-8* (nine patients, 2.0%), and *mcr-4* (one patient, 0.2%). No patient tested positive for *mcr-2, mcr-5, mcr-9*, or *mcr-10*. Overall, 87 (19.3%) of 451 patients were positive for at least one colistin resistance gene.

## Discussion

This study provides important insights into the burden of MDR bacteria and resistance-encoding genes among children under 5 years of age who were hospitalized with acute diarrhea at a provincial pediatric hospital in Vietnam, with 150 (33.2%) of 451 children harboring at least one MDR bacteria and/or one colistin resistance gene. The findings highlight a substantial circulation of MDR organisms in this population, characterized by a high prevalence of ESBL-producing Enterobacterales; the presence of MRSA; and the detection of several clinically significant resistance genes, including *bla_CTX_*_-M_ variants, *bla_TEM_, mecA*, and a frequent carriage of colistin-resistant genes in stool specimens.

In addition, the coexistence of multiple resistance genes within the same individual suggests complex transmission pathways, including household spread, environmental exposure, and health care–associated acquisition. This underscores the importance of strengthening infection control, improving antibiotic stewardship, and enhancing diagnostic capacity at the provincial level. In resource-limited settings such as Vietnam, routine microbiological testing is often unavailable, delaying appropriate antimicrobial therapy and limiting opportunities for surveillance [[20], [21], [22]]. The integration of molecular diagnostics, as applied in this study, is, therefore, essential for early detection of resistance threats.

Nearly 97% of children received antibiotics, with cephalosporins being the most common, despite acute diarrhea being predominantly viral in this setting [17]. This pattern underscores the ongoing challenge of antibiotic overuse in Vietnam, which continues to drive selective pressure for MDR organisms. Studies from other low- and middle-income countries similarly show disproportionately high antibiotic prescription rates for diarrhea in children [[23], [24], [25], [26]]. Over-prescribing also increases the likelihood of colonization with ESBL-producing Enterobacterales, further perpetuating a cycle of antimicrobial resistance. It is important to note that stool samples were collected at admission, before antibiotic initiation. Therefore, the MDR organisms detected in this study reflect preexisting intestinal carriage rather than selection pressure from in-hospital antibiotic use. This finding suggests that children were already colonized with MDR bacteria upon presentation, likely due to previous community exposure, previous health care contact, or antibiotic use before admission.

The predominance of *E. coli* as the most frequently isolated MDR bacteria is consistent with global evidence that ESBL-producing *E. coli* are major contributors to antibiotic resistance in the pediatric gastrointestinal system [[27], [28], [29], [30], [31]]. Importantly, the detection of MDR *E. coli* in stool samples does not imply a causal role in diarrheal disease because these organisms may represent asymptomatic carriage occurring alongside viral or bacterial enteric pathogens identified by molecular methods. The high proportion of isolates carrying the *bla_CTX-M-A_* gene reflects regional epidemiologic patterns reported across Southeast Asia, where CTX-M enzymes have largely replaced older ESBLs, such as *bla_SHV_* and *bla_TEM_*, in the community [32,33]. The detection of a significant proportion of *bla_NDM_* in Enterobacteriaceae isolates is clinically significant given the rapid global dissemination of carbapenem-resistant strains and limited treatment options available to infected patients.

Our findings on the presence of MRSA align with previous reports from Vietnam demonstrating widespread community-associated MRSA circulation [[34], [35], [36]]. These results reinforce the concern that even children without previous hospital exposures may harbor resistant organisms, reflecting the broader community reservoir of resistance.

The emergence of plasmid-mediated colistin resistance has been reported in Vietnam in clinical and environmental settings, likely fueled by the widespread agricultural use of colistin [37,38]. The high prevalence observed in samples collecting at admission supports the notion that colistin-resistant bacteria are widely disseminated in the community and may be acquired through food, water exposure, or person-to-person transmission. Given colistin’s role as a last-resort antibiotic, this finding raises concerns about the future viability of treatment options for severe pediatric infections.

Several limitations should be considered when interpreting the findings. First, the study was conducted at a single provincial pediatric hospital, which may limit generalizability to other regions of Vietnam with different epidemiologic characteristics. Second, only children hospitalized with acute diarrhea were included and the prevalence of MDR bacteria among healthy children or those with other clinical syndromes remains unknown. Third, although molecular methods allowed the detection of key resistance genes, whole-genome sequencing was not performed, which would have provided deeper insights into transmission pathways, clonal relationships, and plasmid structures. Fourth, because this was a descriptive study without a control group, causal relationships between antibiotic exposure and MDR carriage could not be formally assessed. In addition, the absence of quantitative bacterial load measurements and environmental sampling limits conclusions about sources and transmission routes. Finally, a distinction between bacterial carriage and etiologic agents of diarrhea is essential. In this study, MDR bacteria and resistance genes were identified in stool samples collected at admission, whereas the etiology of diarrhea was predominantly viral, as previously reported. Therefore, MDR organisms detected by culture or molecular assays should be interpreted as markers of antimicrobial resistance circulation within the pediatric gut microbiota rather than direct causes of diarrheal illness. This distinction is critical to avoid misinterpretation of the clinical significance of MDR bacteria and guide appropriate antimicrobial stewardship strategies.

## Conclusion

This study demonstrates a substantial burden of multidrug-resistant bacteria among Vietnamese children who were hospitalized with acute diarrhea, with a high prevalence of ESBL-producing Enterobacterales, MRSA, and plasmid-mediated colistin resistance genes. The predominance of *bla_CTX-M_* alleles, significant presence of *mecA*, and notable detection of *mcr-1* and *mcr-3* underscore the complexity and severity of antimicrobial resistance challenges in pediatric populations. These findings highlight an urgent need for strengthened antimicrobial stewardship, rational antibiotic prescribing, improved infection prevention measures, and enhanced diagnostic capacity. Addressing these challenges is essential to protect the effectiveness of current antibiotics and improve clinical outcomes for children in Vietnam and other resource-limited settings.

## Declaration of competing interest

The authors have no competing interests to declare.

## Appendix Supplementary materials

Image, application 1
