# Supplementary material for: Carriage of multidrug-resistant bacteria and encoding genes among Vietnamese children with acute diarrhea
Source: IJID Reg. 2026 Jan 12;18:100844. doi: 10.1016/j.ijregi.2026.100844 (PMC12874097; doi:10.1016/j.ijregi.2026.100844)
Supplement: Supplementary file 1 [file mmc1.docx]

Supplementary table 1. Antibiogram profile of *Staphylococcus aureus*, isolated on MRSA medium (red = resistant, yellow = intermediate, green = sensitive)

| Isolates | Clindamycin | Doxycycline | Erythromycin | Fusidic acid | Fosfomycin | Gentamicin | Oxacillin | Pristinamycin | Rifampicin | Teicoplanin | Vancomycin | Ciprofloxacin | fusidic acid | Sulfamethoxazole–trimethoprim |
| --- | --- | --- | --- | --- | --- | --- | --- | --- | --- | --- | --- | --- | --- | --- |
| *Staphylococcus aureus* |  |  |  |  |  |  |  |  |  |  |  |  |  |  |
| *Staphylococcus aureus* |  |  |  |  |  |  |  |  |  |  |  |  |  |  |
| *Staphylococcus aureus* |  |  |  |  |  |  |  |  |  |  |  |  |  |  |
| *Staphylococcus aureus* |  |  |  |  |  |  |  |  |  |  |  |  |  |  |
| *Staphylococcus aureus* |  |  |  |  |  |  |  |  |  |  |  |  |  |  |
| *Staphylococcus aureus* |  |  |  |  |  |  |  |  |  |  |  |  |  |  |
| *Staphylococcus aureus* |  |  |  |  |  |  |  |  |  |  |  |  |  |  |
| *Staphylococcus aureus* |  |  |  |  |  |  |  |  |  |  |  |  |  |  |
| *Staphylococcus aureus* |  |  |  |  |  |  |  |  |  |  |  |  |  |  |
| *Staphylococcus aureus* |  |  |  |  |  |  |  |  |  |  |  |  |  |  |
| *Staphylococcus aureus* |  |  |  |  |  |  |  |  |  |  |  |  |  |  |
| *Staphylococcus aureus* |  |  |  |  |  |  |  |  |  |  |  |  |  |  |
| *Staphylococcus aureus* |  |  |  |  |  |  |  |  |  |  |  |  |  |  |
| *Staphylococcus aureus* |  |  |  |  |  |  |  |  |  |  |  |  |  |  |
| *Staphylococcus aureus* |  |  |  |  |  |  |  |  |  |  |  |  |  |  |
| *Staphylococcus aureus* |  |  |  |  |  |  |  |  |  |  |  |  |  |  |
| *Staphylococcus aureus* |  |  |  |  |  |  |  |  |  |  |  |  |  |  |

Supplementary table 2. Antibiogram profile of pathogen, isolated on MacConkey medium (red = resistant, yellow = intermediate, green = sensitive)

| Isolates | Amikacin | Amoxicillin–clavulanic acid | Amoxicillin | Ciprofloxacin | Ceftriaxone | Doxycycline | Ertapenem | Cefepime | Fosfomycin | Gentamicin | Imipenem | Sulfamethoxazole–trimethoprim | Piperacillin–tazobactam |
| --- | --- | --- | --- | --- | --- | --- | --- | --- | --- | --- | --- | --- | --- |
| *Enterobacter cloacae* |  |  |  |  |  |  |  |  |  |  |  |  |  |
| *Enterobacter cloacae* |  |  |  |  |  |  |  |  |  |  |  |  |  |
| *Enterobacter cloacae* |  |  |  |  |  |  |  |  |  |  |  |  |  |
| *Escherichia coli* |  |  |  |  |  |  |  |  |  |  |  |  |  |
| *Escherichia coli* |  |  |  |  |  |  |  |  |  |  |  |  |  |
| *Escherichia coli* |  |  |  |  |  |  |  |  |  |  |  |  |  |
| *Escherichia coli* |  |  |  |  |  |  |  |  |  |  |  |  |  |
| *Escherichia coli* |  |  |  |  |  |  |  |  |  |  |  |  |  |
| *Escherichia coli* |  |  |  |  |  |  |  |  |  |  |  |  |  |
| *Escherichia coli* |  |  |  |  |  |  |  |  |  |  |  |  |  |
| *Escherichia coli* |  |  |  |  |  |  |  |  |  |  |  |  |  |
| *Escherichia coli* |  |  |  |  |  |  |  |  |  |  |  |  |  |
| *Escherichia coli* |  |  |  |  |  |  |  |  |  |  |  |  |  |
| *Escherichia coli* |  |  |  |  |  |  |  |  |  |  |  |  |  |
| *Escherichia coli* |  |  |  |  |  |  |  |  |  |  |  |  |  |
| *Escherichia coli* |  |  |  |  |  |  |  |  |  |  |  |  |  |
| *Escherichia coli* |  |  |  |  |  |  |  |  |  |  |  |  |  |
| *Escherichia coli* |  |  |  |  |  |  |  |  |  |  |  |  |  |
| *Escherichia coli* |  |  |  |  |  |  |  |  |  |  |  |  |  |
| *Escherichia coli* |  |  |  |  |  |  |  |  |  |  |  |  |  |
| *Escherichia coli* |  |  |  |  |  |  |  |  |  |  |  |  |  |
| *Escherichia coli* |  |  |  |  |  |  |  |  |  |  |  |  |  |
| *Escherichia coli* |  |  |  |  |  |  |  |  |  |  |  |  |  |
| *Escherichia coli* |  |  |  |  |  |  |  |  |  |  |  |  |  |
| *Escherichia coli* |  |  |  |  |  |  |  |  |  |  |  |  |  |
| *Escherichia coli* |  |  |  |  |  |  |  |  |  |  |  |  |  |
| *Escherichia coli* |  |  |  |  |  |  |  |  |  |  |  |  |  |
| *Escherichia coli* |  |  |  |  |  |  |  |  |  |  |  |  |  |
| *Escherichia coli* |  |  |  |  |  |  |  |  |  |  |  |  |  |
| *Escherichia coli* |  |  |  |  |  |  |  |  |  |  |  |  |  |
| *Escherichia coli* |  |  |  |  |  |  |  |  |  |  |  |  |  |
| *Escherichia coli* |  |  |  |  |  |  |  |  |  |  |  |  |  |
| *Escherichia coli* |  |  |  |  |  |  |  |  |  |  |  |  |  |
| *Escherichia coli* |  |  |  |  |  |  |  |  |  |  |  |  |  |
| *Escherichia coli* |  |  |  |  |  |  |  |  |  |  |  |  |  |
| *Escherichia coli* |  |  |  |  |  |  |  |  |  |  |  |  |  |
| *Escherichia coli* |  |  |  |  |  |  |  |  |  |  |  |  |  |
| *Escherichia coli* |  |  |  |  |  |  |  |  |  |  |  |  |  |
| *Escherichia coli* |  |  |  |  |  |  |  |  |  |  |  |  |  |
| *Escherichia coli* |  |  |  |  |  |  |  |  |  |  |  |  |  |
| *Escherichia coli* |  |  |  |  |  |  |  |  |  |  |  |  |  |
| *Escherichia coli* |  |  |  |  |  |  |  |  |  |  |  |  |  |
| *Escherichia coli* |  |  |  |  |  |  |  |  |  |  |  |  |  |
| *Escherichia coli* |  |  |  |  |  |  |  |  |  |  |  |  |  |
| *Escherichia coli* |  |  |  |  |  |  |  |  |  |  |  |  |  |
| *Escherichia coli* |  |  |  |  |  |  |  |  |  |  |  |  |  |
| *Escherichia coli* |  |  |  |  |  |  |  |  |  |  |  |  |  |
| *Escherichia coli* |  |  |  |  |  |  |  |  |  |  |  |  |  |
| *Escherichia coli* |  |  |  |  |  |  |  |  |  |  |  |  |  |
| *Escherichia coli* |  |  |  |  |  |  |  |  |  |  |  |  |  |
| *Escherichia coli* |  |  |  |  |  |  |  |  |  |  |  |  |  |
| *Escherichia coli* |  |  |  |  |  |  |  |  |  |  |  |  |  |
| *Escherichia coli* |  |  |  |  |  |  |  |  |  |  |  |  |  |
| *Escherichia coli* |  |  |  |  |  |  |  |  |  |  |  |  |  |
| *Escherichia coli* |  |  |  |  |  |  |  |  |  |  |  |  |  |
| *Escherichia coli* |  |  |  |  |  |  |  |  |  |  |  |  |  |
| *Escherichia coli* |  |  |  |  |  |  |  |  |  |  |  |  |  |
| *Escherichia coli* |  |  |  |  |  |  |  |  |  |  |  |  |  |
| *Escherichia coli* |  |  |  |  |  |  |  |  |  |  |  |  |  |
| *Escherichia coli* |  |  |  |  |  |  |  |  |  |  |  |  |  |
| *Escherichia coli* |  |  |  |  |  |  |  |  |  |  |  |  |  |
| *Escherichia coli* |  |  |  |  |  |  |  |  |  |  |  |  |  |
| *Klebsiella pneumoniae* |  |  |  |  |  |  |  |  |  |  |  |  |  |
| *Klebsiella pneumoniae* |  |  |  |  |  |  |  |  |  |  |  |  |  |
| *Klebsiella pneumoniae* |  |  |  |  |  |  |  |  |  |  |  |  |  |
| *Klebsiella pneumoniae* |  |  |  |  |  |  |  |  |  |  |  |  |  |
| *Klebsiella pneumoniae* |  |  |  |  |  |  |  |  |  |  |  |  |  |
| *Klebsiella pneumoniae* |  |  |  |  |  |  |  |  |  |  |  |  |  |
| *Klebsiella pneumoniae* |  |  |  |  |  |  |  |  |  |  |  |  |  |
| *Klebsiella pneumoniae* |  |  |  |  |  |  |  |  |  |  |  |  |  |
| *Klebsiella pneumoniae* |  |  |  |  |  |  |  |  |  |  |  |  |  |
| *Klebsiella pneumoniae* |  |  |  |  |  |  |  |  |  |  |  |  |  |
| *Klebsiella pneumoniae* |  |  |  |  |  |  |  |  |  |  |  |  |  |
| *Klebsiella pneumoniae* |  |  |  |  |  |  |  |  |  |  |  |  |  |
| *Klebsiella variicola* |  |  |  |  |  |  |  |  |  |  |  |  |  |
| *Salmonella Paratyphi* |  |  |  |  |  |  |  |  |  |  |  |  |  |
| *Salmonella Typhi* |  |  |  |  |  |  |  |  |  |  |  |  |  |

Supplementary table 3. Antibiogram profile of pathogens isolated on SMART medium (red = resistant, yellow = intermediate, green = sensitive)

| Isolates | Amikacin | Ticarcillin–clavulanic acid | Ticarcillin | Ciprofloxacin | Ceftazidime | Colistin | Doripenem | Ertapenem | Cefepime | Fosfomycin | Imipenem | Sulfamethoxazole–trimethoprim | Tobramycin | Piperacillin–Tazobactam |
| --- | --- | --- | --- | --- | --- | --- | --- | --- | --- | --- | --- | --- | --- | --- |
| *Escherichia coli* |  |  |  |  |  |  |  |  |  |  |  |  |  |  |
| *Escherichia coli* |  |  |  |  |  |  |  |  |  |  |  |  |  |  |
| *Enterobacter kobei* |  |  |  |  |  |  |  |  |  |  |  |  |  |  |
| *Klebsiella pneumoniae* |  |  |  |  |  |  |  |  |  |  |  |  |  |  |
| *Escherichia coli* |  |  |  |  |  |  |  |  |  |  |  |  |  |  |
| *Escherichia coli* |  |  |  |  |  |  |  |  |  |  |  |  |  |  |
